# Reliability and validity of the Persian version of readiness for inter-professional learning scale

**DOI:** 10.5116/ijme.5da4.37c2

**Published:** 2019-11-04

**Authors:** Marzieh Ataollahi, Mitra Amini, Somayeh Delavari, Leila Bazrafkan, peyman Jafari

**Affiliations:** 1 Clinical Education Research Center, Shiraz University of Medical Sciences, Shiraz, Iran; 2 Evidence Based Medicine Research Center, Endocrinology and Metabolism Clinical Sciences Institute, Tehran University of Medical Sciences, Tehran, Iran; 3 Department of Biostatistics, Shiraz University of Medical Sciences, Shiraz, Iran

**Keywords:** Inter-professional education, validity, reliability, CFA, Iran

## Abstract

**Objectives:**

To assess the validity and reliability of the Persian version of the Readiness for Inter-Professional Learning Scale (RIPLS).

**Methods:**

A cross-sectional study was performed among final-year medical students in Iran. A total of 200 students completed the Persian versions of the RIPLS questionnaire using convenience sampling. To evaluate the construct validity of the RIPLS questionnaire, data were subjected to confirmatory factor analysis (CFA).  Some goodness-of-fit indicators were used to assess the hypothesized model. The hypothesised models were tested with LISREL 7.8.

**Results:**

Cronbach’s alphas for 9 teamwork and collaboration (TAC), 3 negative professional identity (NPI), 4 positive professional identity (PPI) and 3 Roles and responsibilities (RAR) items were 0.89, 0.60, 0.86 and 0.28 respectively. The whole RIPLS was found to be highly reliable (19 items; α= 0.94).  The set of fit statistics show that the hypothesised four-factor model fits the sample data.

**Conclusions:**

The results of the study show that the Persian version of the RIPLS may be a valid and reliable scale. In addition, the results of CFA show that the hypothesised four-factor model appears to be a good fit to the data. However, the Persian version of the subscales of NPI and RAR needs to be developed. The implications and limitations of the study are discussed.

## Introduction

Currently, a variation of pedagogical approaches are used for improving student performance and increasing student achievement.^1^^,^^2^ One of these approaches is Inter-Professional Education (IPE). IPE refers to situations where students from two or more health professions learn from each other to enhance collaborative practice, which in turn promotes health and treats patients.  In addition, IPE allows students to learn cooperation and teamwork in an integrated way to solve the problems of patients in a collaborative team environment.^3^^-^^5^ A considerable amount of literature has been published on IPE. These studies show the importance of IPE in healthcare disciplines. For example, a systematic review shows that IPE experiences improve the knowledge and attitudes of learners towards interprofessional care, communication skills, problem- solving abilities and appropriate interactions.^6^  A further systematic review shows that IPE is associated with a range of positive outcomes in the health care system, such as decreased clinical errors, increased patient care, management, patient satisfaction, and teamwork. However, some studies based on this review showed that the IPE experiences did not affect patient care and practice.^7^ Buring and colleagues reported a piece of further evidence to support IPE. They indicated that IPE enhances teamwork, leadership, competencies and learning outcomes. However, they also highlighted barriers to IPE, such as logistical and resource issues.^8^

Given the importance that IPE has placed on the overall quality of patient care, medical educators have made concentrated efforts to construct scales to assess IPE in different cultures. For example, Lauffs and colleagues used the RIPLS to identify its validity and reliability for Swedish students.^9^ The RIPLS has been widely used in health-professions education as it is a valid and reliable scale for assessing the readiness of students for interprofessional learning.^10^ Because of this, many countries translated and adapted the RIPLS to determine the readiness of their students for IPE.^9^^-^^11^ Along with this growth in IPE, however, there is an increasing concern over the lack of a valid and reliable Persian scale to measure the readiness of students for IPE. Therefore, we felt that it is essential to construct a Persian scale, which fits into a Persian culture. The purpose of this research is to determine whether or not the RIPLS can be adapted for Persian healthcare professions.

## Methods

### Study design and participants

In this study, the methodological approach taken is a quantitative study using a cross-sectional design. Participants consisted of 200 final- year medical students who completed the scale using a convenience sampling approach. Before commencing the study, ethical clearance was obtained from the Shiraz University of Medical Sciences. Before initiating research activities, informed consent from participants was obtained. The confidentiality and anonymity of the data were guaranteed.  We also informed the participants of their right to refuse to participate for any reason without penalty.

### Data collection method

In this study, we used the RIPLS to respond to the purpose of the study raised in the Introduction section. Parsell and Bligh originally developed the RIPLS in 1999.^10^ McFadyen and colleagues further developed the RIPLS into a four-factor model in 2005.^12^ In this study, the Persian version of the RIPLS by McFadyen and colleagues was used. It has been validated and found to be reliable in Sweden,^9^ Germany,^11^ and Japan.^13^ The 19 items of this scale are rated on a 5-point scale ranging from 1= strongly disagree to 5= strongly agree (higher scores show greater the readiness for interprofessional learning). The RIPLS comprises of 4 unconnected subscales: Teamwork and Collaboration (TAC, Items 1-9), Negative Professional Identity (NPI, Items10-12), Positive Professional Identity (PPI, Items 14-16) and Roles and Responsibilities (RAR, Items 17-19).

### Procedure

The McFadyen’s version of the RIPLS was first translated into Persian by two of the authors. The translated version of the RIPLS was reviewed for inconsistencies using the back-translation approach. After resolving the inconsistencies, we distributed the final translated version of the RIPLS to final year medical students. The purpose of the RIPLS was thoroughly described to the students and we explained that we would use the study results anonymously and confidentially for research purposes. We then asked the students to sign a consent form to show that they agreed to participate in the study.

### Statistical analyses

To assess the internal consistency of the RIPLS, we calculated Cronbach’s alpha for each of those above-mentioned subscales. A coefficient alpha greater than or equal to 0.70 would be considered to be satisfactory reliability of the scale score.^14^^, ^^15^ To evaluate the hypothesized four-factor model of the RIPLS, the data were subjected to the Confirmatory Factor Analysis (CFA). The goodness-of-fit statistics resulting from this analysis are reported. They are Root Mean Square Error of Approximation (RMSEA), Normed Fit Index (NFI), Non-Normed Fit Index (NNFI), Comparative Fit Index (CFI), Goodness-of-Fit Index (GFI) and Adjusted Goodness-of-Fit Index (AGFI). The acceptable thresholds of NFI, NNFI, CFI, GFI, and AGFI are greater than 0.95. The value less than 0.07 for RMSEA indicates the fit index.^16^ The reliability of the subscales of the RIPLS also reported. LISREL 7.8 software and SPSS 21 were used to analyze data.

## Results

### Examination of internal consistency

Cronbach’s alpha was used to examine the internal consistency of the items within each subscale.  The results showed that the Cronbach’s alpha coefficients for the TAC, NPI, PPI and RAR subscales were 0.89, 0.60, 0.86 and 0.28 respectively. Accordingly, the items in the two subscales (TAC and PPI) have satisfactory internal consistency, and their alpha exceeds the 0.70 threshold value (Table 1).

**Table 1:** Cronbach's alpha, Mean and Standard Deviation of the subscales of the RIPLS

Subscale	No. Items	Cronbach's alpha	Mean (SD)
TAC	9	0.89	1.96 (0.5)
NPI	3	0.60	2.60 (0.73)
PPI	4	0.86	2.00 (0.57)
RAR	3	0.28	2.39 (0.57)

**Figure 1:**
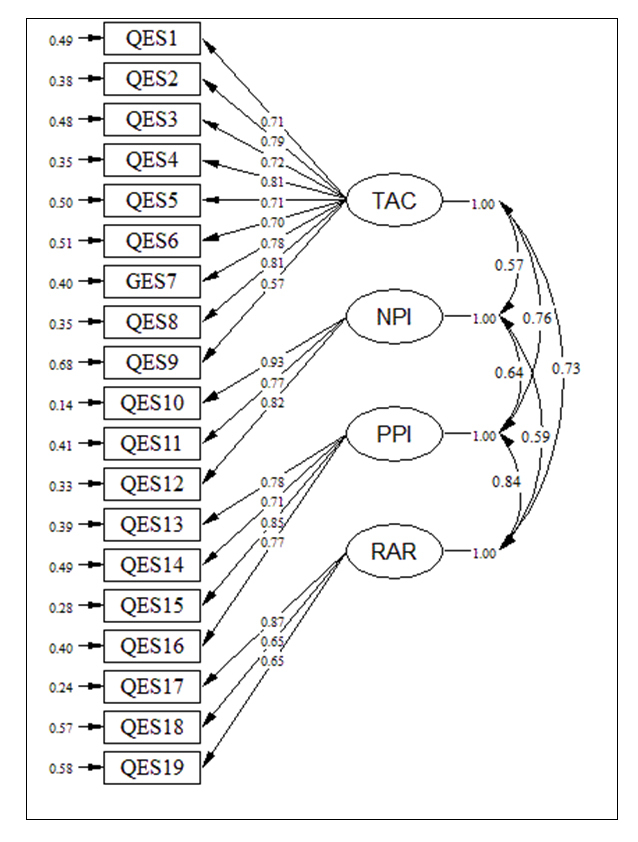
The results of the path analysis for the hypothesized model. Standardised coefficients are presented

Figure 1 shows each item has a loading (the standardized regression coefficient) corresponding to each of the four subscales. The numbers “1” in the diagram show that the factor loading regression coefficient has been fixed to 1.  Errors of measurement associated with each item are seen in Figure 1. As we can see from the path diagram, the correlation between the loading estimates and the subscales are acceptable, indicating the data fit the hypothesised model (Figure 1). The results of the CFA showed in Table 2.

In reviewing the goodness-of-fit statistics in Table 2, we can see that the factor model fit the data almost equally well. There are no outstanding fit statistics values suggestive of the four-factor model misfit, except GFI.

**Table 2:** The results of the CFA for the hypothesised models

Threshold	Fit indexes obtained	Fit index
<0.07	0.06	Root Mean Square Error of Approximation (RMSEA)
>0.95	0.95	Normal Fit Index (NFI)
>0.95	0.96	Non-Normed Fit Index (NNFI)
>0.95	0.97	Comparative Fit Index (CFI)
>0.95	0.94	Goodness for Fit Index (GFI)
>0.95	0.99	Adjusted Goodness for Fit Index (AGFI)

## Discussion

This study aimed to investigate whether the 4‐factor model from the original version of the RIPLS could be applied to the Persian translation of the scale. Because of this, the original version of the RIPLS was translated into the Persian language in order to identify its validity and reliability among medical students at Shiraz University of Medical Sciences.

The value of RMSEA suggests that the 4-factor model fits the population covariance matrix. It is a very useful fit index because of “its sensitivity to the number of estimated parameters in the model”.^16^  In our study, the CFI, which is usually estimated for checking goodness-of-fit a statistical model, is greater than the threshold (0.95) suggesting the 4-factor model fits an independent or null model. The GFI, which is analogous to R2 in multiple regression, indicates the proportion of variance in the sample explained by the estimated population covariance.^17^ The GFI is slightly less than the cut-off for a good fit. However, the AGFA indicates the 4-factor model fits the data. This suggests the 4-factor model needs to be cautiously applied for medical students. Finally, the NFI and NNFI were estimated to measure the disparity between the χ^2^ value of the 4-factor model to the χ^2^ value of the independent or null model. The values of the NFI and NNFI are indicative of a good –fitting model. Given that the values of goodness-of-fit statistics, it seems that the 4-factor model fits the sample data, although the two indices are a little below the threshold. Additional studies are necessary in order to adapt the 4-factor model for Iranian medical students. Furthermore, an inspection of the correlation between the loading estimates and the subscales in the path diagram, show the data fit the 4-factor model.

Cronbach’s alphas for the coefficients of the subscales TAC, NPI, PPI, and RAR in the Persian and other versions were approximately similar (0.89, 0.60, 0.86 and 0.28).^9^^, ^^11^^, ^^18^^, ^^19^ The low value of the RAR subscale could be due to the fact that the number of items is small. The key strength of this study is the high response rate from a convenience sample, and a number of important limitations need to be considered. First, this study was only conducted in a single institution; therefore, the generalisability of this study is limited.  Second, the study sample only consists of medical students and they may not be representative of other allied health students. Third, social desirability response bias may occur due to the nature of self-reports.  These study limitations suggest that further data collection is required to culturally adapt a Persian translated version of the readiness for interprofessional learning scale (RIPLS).

## Conclusions

Taken together, the results of this study make several contributions to the current literature.  They suggest that the Persian translated version of the RIPLS may be a valid and reliable tool for assessing the attitudes of medical students in Iran. However, further research needs to need to be done to examine the subscales RAR and NPI before the scale is adapted for Persian healthcare students.

### Acknowledgments

The authors thank all students who contributed to this study.

### Conflict of Interest

The authors declare that they have no conflict of interest.
